# On Inflammation of the Uterus, and Induration and Ulceration of the Neck of That Organ

**Published:** 1857-01

**Authors:** Geo. W. Garland


For the N. H. Journal of Medicine.

### *On Inflammation of the Uterus, and Induration and Ulceration of the Neck of that Organ.* By Geo. W. Garland, M. D.

I design my remarks shall be as brief as possible, and wholly practical. An accession of a single practical fact, or well established principle, is truly appreciated by the profession and as my remarks are gatherings from practice and observation, I submit them to the profession believing some one may be benefitted by the hints they contain.

The uterus, like every other organ in the human body, is subject to various degrees of inflammation, from the slightest to the most intense, from that indicated by an augmented secretion of mucus from its liming membrane, to that involving its entire substance and which runs rapidly to gangrene.

As I wish mainly to refer to some of the events of *metritis*, I hall not follow the form of a treatise, because all that I could say of the anatomical structure of the uterus, its inflammation, symptoms, &c., can be found elsewhere. Neither shall I refer to all the causes of inflammation of the womb, many of which are common to the phlegmasia in general, while others are peculiar to the uterus and vagina;—as the first, sexual connection,—suppression of the menstrua,—solitary enjoyment,—employment of violent emmenagogues,—severe labor,—manipulations with the hand,—use of instruments, &c. An accurate account of these as well as the symptoms of metritis can be found by referring to any of our standard works on diseases of women. There is one symptom, however, to which I wish to call particular attention, viz: when inflammation has succeeded labor, *the discharge of "sanguineous mucus,"* is *prolonged beyond* the usual period. As a passive inflammation of the uterus may and often does exist, and no marked symptoms manifested, this symptom is of great value in leading to proper investigation.

When the whole organ is inflamed the symptoms are always extremely serious and sufficiently apparent, but when the inflammation is seated in the mucus membrane of the cervix the pain is very slight, if any; or when a small part of the neck is the seat of the inflammation, the pain is at the bottom of the vagina, and does not call our attention to the uterus; hence the importance of this symptom.

I have known a sense of weight in the pelvis to be the only symptom complained of, except this discharge, when on examination per vaginum, the uterus was found congested and twice its natural size and weight; hence the importance and necessity of making inquiries when the sanguineous discharge is prolonged.

Whether the inflammation or congestion of the uterus is general or partial, there is, when compared with other organs, an unusual tendency to end in induration. This can only be accounted for by the anatomical position of the womb, by the crowded state of the parts when it is enlarged, by its pendulous position, and the gravitating tendency of its circulation, all of which tend to prevent its blood-vessels from returning to their normal state, after having been largely distended; and also accounts for the feeble action of its absorbant vessels.

That induration of the uterine neck, and ulceration about the os, is more frequently met as a sequela of inflammation, than what is found in other parts of the body I am fully convinced. My own observation has again and again admonished me of the necessity of making examinations by the touch, and with the speculum, during and after symptoms of uterine inflammation, from the fact that induration and ulceration, the legitimate fruit of inflammation, are quite sure to follow if the congestion, which is always left after the acute inflammation subsides, is not removed.

I visited a lady to-day, who cannot stand or walk, without suffering intensely from pelvic weight and pain in the back. She had a laborious labor, three months since, which was followed by a sanguineous discharge for six weeks, and has had leucorrhœa constantly since that discharge ceased. Examined per vaginum—found the uterus large and low in the vagina—neck inflamed—os open, so as to admit the index finger to the first joint—lips thick, hard and uneven—leucorrhœa mostly uterine. Physicians familiar with female complaints will recognize in this short account, an example of a large majority of cases, to which they are called and consulted by patients, who are troubled with "falling of the womb."

I have treated many cases of general and partial inflammation of the uterus, with a view to restore that organ to its normal condition and I have nothing *new* to add to the treatment recommended by our authors, but would more *urgently* recommend *free leeching* of the cervix in all cases unless some constitutional condition forbids depletion.

During the last few years, I have used leeches in four cases of metritis, succeeding labor, and several cases produced by other causes, and the results have been equal, to say the least, to that obtained by leeching other parts. I most confidently recommend free leeching, tepid emollient injections, and subsequently cold astringent injections, in all cases of inflammation of the uterus; and that the progress of the disease be watched and the treatment *continued until the organ has regained its natural size and weight.* Unless this is faithfully attended to, nine out of every ten cases will, in from three months to one year, be followed with induration of the neck and perhaps ulceration about the os.

I would not deny that induration and ulceration may be produced by simple congestion, without active or passive inflammation. Dr. Tilt and others assert that the neck of the uterus possesses an erectile tissue, which becomes, by the various stimuli to which it is exposed exaggerated to engorgement. We know that parts possessed of this tissue do not recover from congestion as readily as parts not so organized. Still, I have never seen a case where positive evidence of previous inflammation could not be gathered.

Examinations with the speculum during the progress of metritis reveals the true state and stage of the disease, and will establish the accuracy of the following remarks.

Engorgement is the common, indeed, almost universal condition of any part or organ, which has suffered from recent inflammation. The uterus, as I have stated, is, from its position, anatomical structure, &c., remarkably liable to engorgement. This, when not removed by treatment, terminates in hypertrophy, induration and ulceration of the neck and os.

An afflux of blood to any part destroys the balance between nutrition and absorption, and induration frequently follows from an undue accumulation of fluids, from the presence in the internal texture of parts, in the little spaces existing between their component tissues of fluid or solid matters or both, which are not found there in a healthy state. Blood, or fluids from the blood, may fill and wholly obliterate the interstices and concreting, tend to solidify and harden the part which they occupy. In technical phrase, fluids after having been extravsaated, pass into a solid form, changing the size, form, weight and function of parts; this is induration, the consequence of inflammation. It often plays a fearful part in disorganizing the bodily frame, and in no part of the human organism are its consequences more deplorable than in the organ under consideration.

A lady marries at the age of twenty in the full vigor of health; is soon seized with leucorrhœa and other vaginal and uterine symptoms, such as tenderness of the parts, painful and disagreeable coition, frequent desire to micturate, &c. This state of things will last indefinitely, for months or years, when sooner or later she begins to feel pelvic weight and "dragging pains." No one who has made himself acquainted with the history of diseases of the uterus, can mistake the meaning of these symptoms.

Soon after marriage she was attacked with inflammation of the mucus covering of the vagina, and neck of uterus. Receiving no check by treatment, the cervix became the seat of chronic inflammation. The organ gradually increased in size and weight, by the interstitial deposit referred to above. The uterus which merely floats as it were in the pelvis by its increased weight, gradually overcomes the resistance offered by the vagina and its attachments to the surrounding parts, and descends lower and lower until it reaches the vulva. This is prolapsus uteri, and its usual progress, the time occupied by this change depending on the habits and condition of the patient.

Prolapsus uteri is an accident that does not occur at once and in my opinion it can be prevented in nine cases of ten by treatment, that is, when it is caused by uterine congestion. As soon as the uterus can be brought to its natural size and weight, it will gradually return to its original position, and all symptoms of prolapsus will disappear.

I could refer to many cases which have occurred in my practice, which might be interesting and substantiate the truth of the above statements, but will select those presenting the most points of interest.

June 10th, 1852. I was called to visit Mrs. ——, married, æt. 42. She was before marriage, of an excellent constitution, and always enjoyed perfect health until after her first and only confinement, which took place at the age of 22. She spoke of her labor as having been most severe, and on first "getting round," she experienced a sense of pelvic weight, and bearing down on walking and after any exertion. She recollected distinctly of having had a sanguineous discharge for weeks after getting about, that all who saw her exclaimed, "how very pale you are!" This prolonged show was followed by a profuse leucorrhœa. This condition of health went on for four years, when one day, after putting up window curtains, in doing which she had occasion to change from a chair to the floor, and back again many times, she felt the womb fall quite down to the vulva. She was in considerable pain and sent for her physician, who made an examination and told her she had falling of the womb, and caused her to keep in bed for a week or two. On leaving her bed the same accident occurred and continued to occur subsequently, to the time when I saw her, whenever she attempted exercise.

Her catamenia were menorrhagic and painful. She was very *pale, weak and nervous.* Had not left her house for fifteen years, and for nine years had not taken steps enough per day to cross a common room. She had secured the advice of many physicians as well as every quack that came that way. Absolute quiet, and weeks and months confinement to the bed, with vaginal injections, tonics, &c., had been used with little benefit. For several years the least exertion or attempts at walking would be followed by a show sometimes quite profuse.

On making an examination per vaginum, I found the cervix low down in the vagina, and very voluminous. A medium sized speculum exposed only a part of the cervix. Completely surrounding the os, and extending beyond what might be called the *lips*, a crop of peculiarly delicate granulations stood out, bleeding on the slightest touch but not very sensitive. The entire neck had a livid violet hue. Just within the os were a few points of ulceration. The os was dilated to the size of a ten cent *bit.*

Nitrate of silver was applied to the whole field of granulations, the pencil being passed slowly over the part. The velvet like growth shrank and shriveled before the caustic, bleeding at different points. She was requested to lie in bed, and the following day to use an injection of zinci sul. ʒi to the pint of water. In three days the granulations were much broken down and divested of their smooth heads. On the following day a profuse hemorrhage occurred, which relieved the surrounding congestion so much that instead of the violet hue the part was quite pale. Nitrate of silver was used as before, but less thoroughly. Ordered wine of iron in drachm doses three times a day. Saw her again in four days; found the granulations quite taken away and the surface somewhat tender to the touch. The application of the Nitrate at this time caused heat and smarting which lasted several hours. Iron to be continued.

To shorten the account of this case, I will say the caustic was applied once a week for four weeks, when the ulcers had healed perfectly. The congested vessels about the neck and os, had regained their former size and the uterus seemed one half less in volume and weight. The patient had gained flesh, color and strength, and was filled with hope and a vigorous determination to get well.

She was then ordered exercise in the open air and to use the following injection: zinci sul. ʒij, acet. plumbi ℥ss, aqua bul. O iss, to be used with a block-tin four ounce, female syringe, with ivory tipped nozzle, which, by the way, is the very best female syringe now in use, as from its size and form the patient can give the parts a shower bath, with a force that completely washes away the secretions leaving no coagulated mucus in the vagina to cause irritation as is the case with the common glass instruments.

It will be seen that the profuse bleeding which occurred during the application of the nitrate, completely obviated the necessity of any other local depletion, and at the same time most emphatically established the benefit of local depletion in uterine congestion.

One year since I treated a granulating ulcer of the anterior lip of the os uteri, by the application of caustic to the ulcer, astringent injections, alteratives internally, rest, &c., for five weeks, without any marked improvement. The lips were slightly œdomatous which caused me to delay depletion. Finding the ulcer did not improve, four leeches were applied near the os. They filled well and the bites bled freely. The nitrate of silver was continued and in twenty-one days the ulcer was healed perfectly. I then introduced one of Hodge's pessaries which at once relieved the patient of pelvic weight and pain which had been a constant annoyance for two years.

I do not doubt, recent cases of inflammation of the cervix uteri have been treated successfully by attention to the general health, vaginal injections, hip baths, &c., but we all know the remarkable tendency of inflammation of the uterus to perpetuate itself indefinitely. The granulations met with about the os uteri are precisely like a granular state of the conjunctiva and may often be removed by constitutional treatment as recommended by that truly wonderful man, Dr. Abernethy, in his "Constitutional Treatment of Local Diseases." The medical profession are ready to bear witness to the truthfulness of his unique lectures, and yet we have all seen cases where constitutional treatment, though judiciously administered has failed to cure local diseases.

To successfully combat disease, we require *all* the means at our command, and in recommending local treatment in uterine diseases I would not disparage or cripple any of the resources of our art.

It sometimes happens, after an ulceration has healed, there remains an induration of the neck, and if left to *nature* the ulcer will reappear when the patient returns to her accustomed duties. So long as induration remains it will be vain and injudicious to encourage the patient that she will rid of her trouble. I had a case of this kind the last summer, which I pronounced cured, who returned to me again after a few weeks with all her usual symptoms. On examination with the speculum I found an ill-conditioned ulcer secreting pus abundantly. In such cases the ulcer is secondary to induration and is of minor importance; unless the induration can be removed it is folly to predict a cure.

In the above case, I applied four leeches to the cervix, taking care that they fixed in or near the ulceration. They filled in much less time than is usually occupied, and bites bled rapidly for several hours. I should think one pint of blood escaped from the bites. Saw her in the morning and after washing away the clots and secretions the cervix appeared pale and blanched. The nitrate was then applied. This was the ninth of June. On the twentieth three more leeches were applied, and their bites bled abundantly. Nitrate as before, every third day. At the end of a week the cervix was much reduced in size and high in the vagina; much of the pelvic weight was relieved and also a peculiar irritation at the neck of the bladder, of which she had made great complaint. She was then put upon the following treatment: rest, astringent injections, weekly cauterizations of the os and neck with nitrate of silver, an occasional saline purgative, light diet, and cold shower baths to the loins night and morning. Under this treatment the patient recovered.

It also frequently happens that an obstinate uterine leucorrhœa remains after all symptoms of inflammation and congestion have been subdued. In such cases I have for the last few years, been in the practice of injecting the uterus. At first I used a gum elastic tube connected with a syringe, but as I found some difficulty in introducing through the neck a flexible instrument, I procured a whale-bone nozzle, four inches in length, which being attached to a common syringe, renders the operation perfectly easy without a speculum. The injection should be slightly astringent at first as the immediate effect is sometimes really frightful. If the uterus is irritable, an injection will cause immediate uterine pain, with which the system sympathizes, causing the same symptoms observed in injuries of the testes, and if the injection is very stimulating, the effect will frighten the patient and friends, and although no harm may be done, the physician will find it difficult to secure a repetition of the operation. From one half to one drachm of sul. zinc. to the pint of barley water is the usual strength. I have known a few (three or four,) uterine injections, remove a troublesome leucorrhœa ; and two instances where repeated injections into the uterine cavity, of barley-water, made astringent by adding one drachm of sulphate of zinc to the pint of water, has proved a remedy for sterility.

Mrs. O—— consulted me for a profuse leucorrhœa. Had been married twelve years. Had worked in Lowell Mills previously, and was then troubled with profuse menstruation and leucorrhœa. Since her marriage, she had grown thin, pale, and weak. The leucorrhœa was uterine. I injected the uterus three times a month for three months, giving at the same time, metallic iron (reduced by hydrogen) internally. At the end of this time all leucorrhœal discharges disappeared. In five months from the dates of her first prescription she became pregnant, for the first time at the age of thirty-seven, and in due time was delivered of a fine boy, much to the joy of an affectionate husband. Mrs. O. was his second wife, her husband having a daughter by his first wife, then some twenty years old ; proving beyond a doubt to my mind, that the want of offspring depended upon a disease of the lining of the uterine cavity, and not upon a disease or radical defect of the ovarian glands.

I beg the candid consideration of the readers of the N. H. Journal of Medicine, on this interesting subject and their lenient judgment of my hastily written remarks.

*Lawrence, Mass.*, Sept. 21, 1856.

